# New Operation for Prolapsus Ani

**Published:** 1841-02

**Authors:** 


*New Operation for Prolapsus Ani.* By M. Robert—Relaxation of the sphincter ani being the cause of this disease, all the remedies hitherto employed for its cure are inefficacious when it arrives at the last stage, as they can only act on the mucous membrane of the rectum. These reflections have induced M. Robert to shorten the sphincter in proportion to the amount of relaxations, so that the two cut surfaces of the muscle might unite and form a narrow ring to oppose to the descent of the mucous membrane. This operation was performed with success on a washerwoman, thirty-three years of age, in June, 1839, in the hospital of La Pitié. This woman in her third pregnancy had a prolapsus ani, which was only temporary though it caused some pain. Her fourth pregnancy produced a prolapsus uteri, a permanent and considerable prolapsus ani, with relaxation of the abdominal walls. M. Robert excised a portion of the mucous membrane with some temporary relief; but the disease afterwards increased, the discharge of fæces became involuntary, and she suffered from pains in the loins and upper part of the thighs. When she entered the hospital the sphincter was so much relaxed that four fingers could be easily introduced.

The patient having been prepared for the operation by progressive diminution in diet, and the use of opium in order to effect long-continued constipation, M. Robert proceeded to operate in the following manner:—An incision was made on each side of the anus, each incision being commenced a few lines external to the orifice, and carried backwards towards the coccyx. The fold of integument between the incisions, together with the portion of sphincter it covered, were removed, and the muscle was thus shortened by half its length. The wound was united from one side to the other by three points of suture. On the sixth day after the operation the sutures were removed. Union was nearly complete, but a fistulous passage remained from the anus to the coccyx. On the fifteenth day the woman had not passed any fæces. On the next day the want of defecation being felt, in order to prevent any straining, the bowels were relieved by the curette. On the forty-first day the patient, who before the operation could not retain her fæces, kept an injection during the whole day; there was no more prolapsus; the opening had become of the ordinary size; but the finger when introduced did not experience the energetic contraction of the sphincter which occurs in the normal state. With the exception of a slight protrusion of mucous membrane the cure was complete in August.—*British and Foreign Med. Rev.* Oct., 1849, from *Gaz. Med. de Paris,* June 20, 1840. *Ibid.*

